# The National Outcomes Evaluation Programme in Italy: The Impact of Publication of Health Indicators

**DOI:** 10.3390/ijerph191811685

**Published:** 2022-09-16

**Authors:** Paola Colais, Luigi Pinnarelli, Francesca Mataloni, Barbara Giordani, Giorgia Duranti, Paola D’Errigo, Stefano Rosato, Fulvia Seccareccia, Giovanni Baglio, Marina Davoli

**Affiliations:** 1 Department of Epidemiology, Lazio Regional Health Service, 00147 Rome, Italy; 2 Research and International Relations Unit, Italian National Agency for Regional Healthcare Services (AGENAS), 00187 Rome, Italy; 3 National Centre for Global Health, Istituto Superiore di Sanità, 00161 Rome, Italy

**Keywords:** healthcare services, comparative outcome evaluation, health information system, hospital data, orthopedic, cardiology, gynecology, breast surgery

## Abstract

In Italy the National Outcomes Evaluation Programme, (P.N.E.) is the most comprehensive comparative evaluation of healthcare outcomes at the national level. The aim of this report is to describe the P.N.E. and some of the most relevant results achieved. The P.N.E. analysed 184 indicators on quality of care in 2015–2020 period. The data sources are the Italian Health Information Systems. The indicators reported were: proportion of surgery within 2 days after hip fracture in the elderly (HF), 30-day mortality after hospital admission for acute myocardial infarction (AMI), proportion of reoperations within 90 days of breast-conserving surgery and proportion of primary caesarean deliveries. Risk adjustment methods were used to take into account patients’ characteristics. From 2010 to 2020 the proportion of interventions within 2 days after HF increased from 31.3% to 64.6%, the AMI 30-day mortality decreased from 10.4% to 8.3%, the proportion of reinterventions within 90 days of breast-conserving surgery decreased from 12.0% to 5.9% and the proportion of primary caesarean deliveries decreased from 28.4% to 22.7%. Results by area of residence showed heterogeneity of healthcare quality. We observed a general improvement in different clinical areas not always associated with a reduction of heterogeneity among areas of residence.

## 1. Introduction

Public disclosure of clinical performance of the healthcare providers is becoming increasingly common among the healthcare systems worldwide [1,2,3], and there is evidence of effectiveness of publication of outcome and process indicators in improving quality of health care [4,5,6,7,8].

To promote the dissemination of the results, public and private organisations have published outcome and process indicators in the form of web-based reports to compare healthcare providers’ performance and quality [9,10,11].

In Italy, national and regional research programmes on outcomes have been conducted. The Mattoni-Outcome Project [12] and the subsequent Progr.Es.Si. Project, led by the National Institute of Health, were funded by the Italian Ministry of Health and conducted at national level; the Regional Outcomes Evaluation Programme (P.Re.Val.E.) [13] was conducted in the Lazio region. These programmes evaluated outcome and process indicators in different clinical and surgical areas and were the starting points for the National Outcomes Evaluation Programme, called P.N.E. [14]. P.N.E. management was entrusted to the Italian National Agency for Regional Healthcare Services (AGENAS) by the Italian Ministry of Health. This is the most comprehensive comparative evaluation of healthcare outcomes at the national level, and the only Italian programme to publicly disclose performance data (published online at https://pne.agenas.it/, accessed on 10 June 2022).

After the publication of the first edition of P.N.E. in 2010, a process of improving the quality of care started in Italy; the milestones of this process are summarised in Table 1.

The aim of this study is the evaluation of the impact of publication of outcomes and process indicators at national level as part of P.N.E. The P.N.E. indicators were defined and calculated in order to:

- compare the outcomes of health care provided by different hospitals or in different geographical areas;
- monitor trends in health care quality over time;
- identify critical areas in which to implement programmes that improve health care quality;
- promote internal and external auditing.

This report describes the P.N.E. programme, including information sources, statistical methodologies and, as an example, some of the most relevant results achieved after the publication of the programme in 2010.

## 2. Materials and Methods

### 2.1. Study Design and Data Sources

The P.N.E. programme annually calculates and publishes an increasing number of indicators on quality of care (currently 184, of which 164 relating to hospital care and 20 relating to avoidable hospitalization and outpatient care outcomes). Several clinical areas are covered: cardiovascular, cerebrovascular, digestive, infectious, mental, musculoskeletal, oncologic, otorhinolaryngologic, paediatric, obstetric, respiratory and urologic [14].

Different types of indicators are calculated: health outcome indicators, such as 30-day mortality after an episode of myocardial infarction; process indicators for which an association with improved health outcomes has already been proven, such as intervention within 2 days of hospital admission for hip fracture in the elderly. Furthermore, hospitalization rates for conditions generally treated out of the hospital, such as diabetes, asthma and influenza, are considered an indication of primary care failure [15]. Almost all indicators were selected based on their previous use in international and national studies [9,10,11,12,13,16,17,18,19,20,21,22], while other indicators were conceived in order to describe particular components of care or clinical pathways. Each indicator is calculated following a detailed operative protocol. The Hospital Information System (HIS), the Emergency Information System (EIS) and the Tax Register represent the data sources used in the Programme [14].

Hospital discharge forms from all Italian hospitals are routinely collected by the HIS and EIS; the collected data contain patient demographic data (gender, age), admission and discharge dates, principal diagnosis at discharge and up to 5 secondary diagnoses (according to the International Classification of Disease, 9th Revision, Clinical Modification [ICD-9-CM]), medical procedures or surgical interventions (up to 6), access and discharge from emergency department and status at discharge (alive, dead, transferred to another hospital).

Moreover, the National Tax Register is used to collect information on life status of all patients, including those who died out-of-hospital. HIS records are linked with National Tax Register records using a deterministic record-linkage.

### 2.2. Study Population

The last Edition of P.N.E. analysed hospital discharges in Italy in the 2015–2020 period [15]. Most of the indicators are expressed as ratios: the numerator represents the number of patients with a given outcome (i.e., short-term mortality, hospitalization for specific conditions, the number of treatments/interventions, etc.) and the denominator represents the group of patients at risk.

Some other indicators are expressed as survival/waiting time (e.g., time to intervention for surgery after tibia and fibula fracture). The analyses were performed annually by hospital and by area of residence (local health unit or province of residence) regardless of the hospital in which the patient was treated.

### 2.3. Definition and Attribution of Outcome

The outcomes under study were: 30-day mortality, short-term re-hospitalization, surgical procedures, short-term complications of specific interventions, and waiting times. The outcomes were dichotomous variables and were attributed to the hospital where the first admission occurred and to the patient’s area of residence.

### 2.4. Coexisting Medical Conditions

Risk factors potentially associated with the outcomes under study were chosen among the conditions identified in the literature [9,10,12,15,16,17,19,20,23,24,25,26]. Each patient’s chronic conditions were identified through the specific ICD-9-CM codes registered both at the index hospital admission and at the hospitalizations occurring in the previous 2 years.

For each indicator, the operative protocol including ICD-9-CM codes used to retrieve chronic coexisting conditions is available online at https://pne.agenas.it/ (accessed on 10 June 2022).

### 2.5. Statistical Analysis

Statistical analyses were performed by hospital as well as by area of residence. Risk adjustment methods were used to take into account patients’ characteristics, such as age, sex, disease severity and/or comorbidities, that could be heterogeneously distributed across the hospitals/areas of residence [27].

The risk adjustment models were specific for the study population and allowed to calculate adjusted outcome measures for comparison between hospitals or areas.

Logistic regression models were used for dichotomous outcome variables and survival models for time to event outcomes

For each dichotomous outcome under study, multivariate logistic regression models without interception and centred covariates were applied to estimate adjusted group-specific (hospital/area of residence) log odds of outcome.

Adjusted risks were obtained for each group by back-transforming parameter estimates with the following formulas:

(1) $$Adj\;risk=\left[\frac{\exp\left(estimate\right)}{1+\exp\left(estimate\right)}\right]*k$$

where *k* is a correction coefficient introduced to take into account the nonlinear nature of the logistic model.

*k* is calculated as follows:

(2) $$k=actual\;number\;of\;events/\overset{m}{\underset{j=1}{\sum }}p_{j}*n_{j}$$

where *p_j_* is the adjusted proportion, *n_j_* is the group size, and *m* is the number of groups.

This approach enabled to compare the outcome of a given hospital/area of residence with that of all the other hospitals/areas and with the entire population under study [17,28,29].

The adjusted RR estimated for each hospital/area of residence, the adjusted risk or median waiting time, and the corresponding *p*-value were reported on-line in tabular and graphical forms.

For each indicator, trend analyses from 2010 to 2020 were calculated by hospital and area of residence.

Furthermore, for some indicators, maps of adjusted proportions of outcomes were produced to compare each area of residence (ArcGIS 9.2 software, ESRI, Redlands, CA, USA). The classes used in the maps have been calculated applying the Jenks natural breaks optimization algorithm, which reduces the variance within classes and maximizes the variance between classes [30]. Finally, cross-classified logistic multilevel models were performed to analyse geographic variations [31]. The variance components were expressed in terms of Median Odds Ratios (MORs) [32].

The level of statistical significance was set at 5% (*p* < 0.05), and all analyses were performed using SAS Version 9.4 (SAS/STAT software, version 8. 1999, Cary, NC, USA: SAS Institute, Inc.).

### 2.6. Indicators

For the purpose of this report, results concerning the following indicators were reported: Proportion of surgery within 2 days after hip fracture in the elderly (HF), 30-day mortality after hospital admission for acute myocardial infarction (AMI), Proportion of reoperations within 90 days of breast-conserving surgery and Proportion of primary caesarean deliveries.

## 3. Results

**Figure 1:**
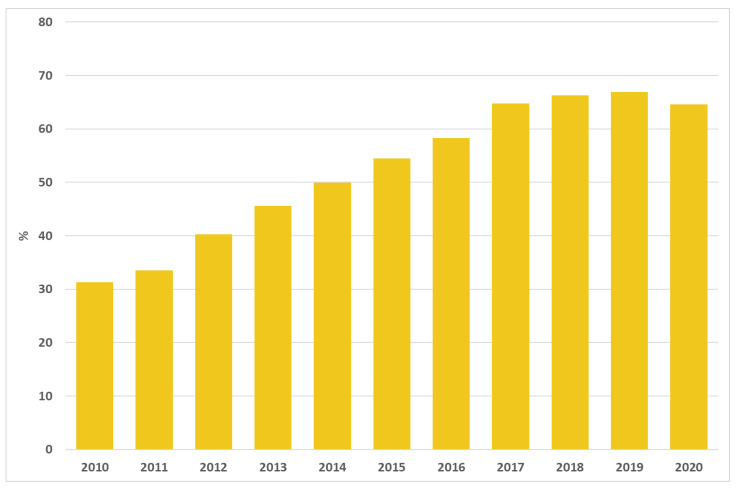
Proportion of surgery within 2 days after hip fracture in patients over 65 years of age. Italy, 2010–2020.

A total of 74,323 admissions for HF were recorded in 2020 in Italy; the proportion of interventions performed within 2 days highly increased in the last few years, from 31.3% in 2010 to 64.6% in 2020 (Figure 1).

**Figure 2:**
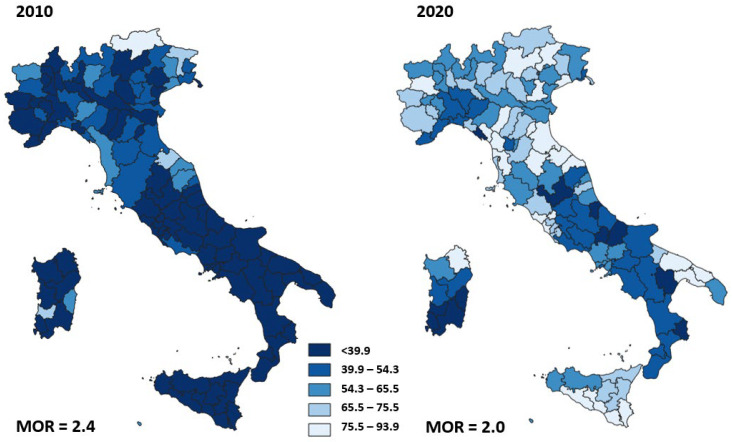
Adjusted proportion of surgery within 2 days after hip fracture in patients over 65 years of age, for Italian area of residence in 2010 and 2020.

Figure 2 shows the adjusted proportion of surgery within 2 days for each area of residence in 2010 and 2020. The geographic variation of proportion of surgery within 2 days ranged from 3.8% to 79.3% (MOR = 2.4) in 2010 and was higher than the geographic variation in 2020, ranging from 19.9% to 93.9% (MOR = 2.0).

**Figure 3:**
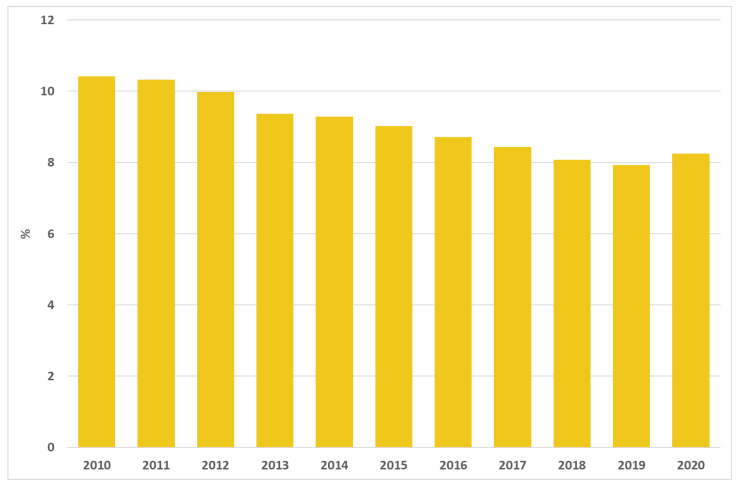
30-day mortality rates after acute myocardial infarction. Italy, 2010–2020.

In 2020, 75,433 AMI episodes were recorded in Italy; the 30-day mortality rates after AMI decreased in the last few years, from 10.4% in 2010 to 8.3% in 2020 (Figure 3).

**Figure 4:**
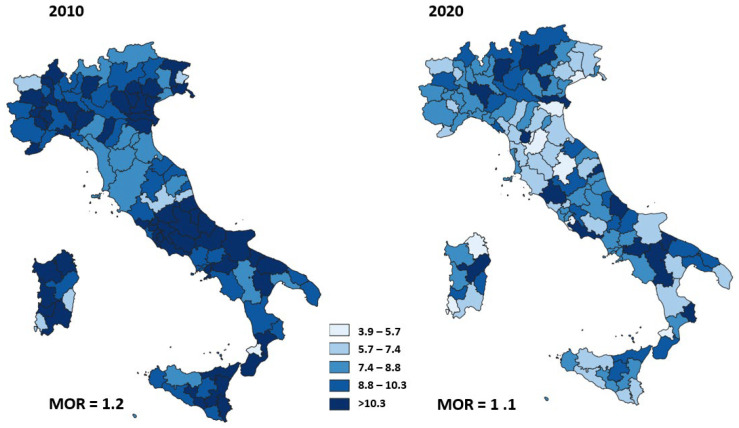
Adjusted 30-day mortality rates after acute myocardial infarction for Italian area of residence in 2010 and 2020.

Figure 4 shows the adjusted 30-day mortality rates after AMI according to area of residence in 2010 and 2020. The geographic variation of AMI mortality ranged from 5.6% to 16.5% (MOR = 1.2) in 2010 and was similar to the geographic variation of AMI mortality in 2020, ranging from 3.9% to 15.1% (MOR = 1.1).

**Figure 5:**
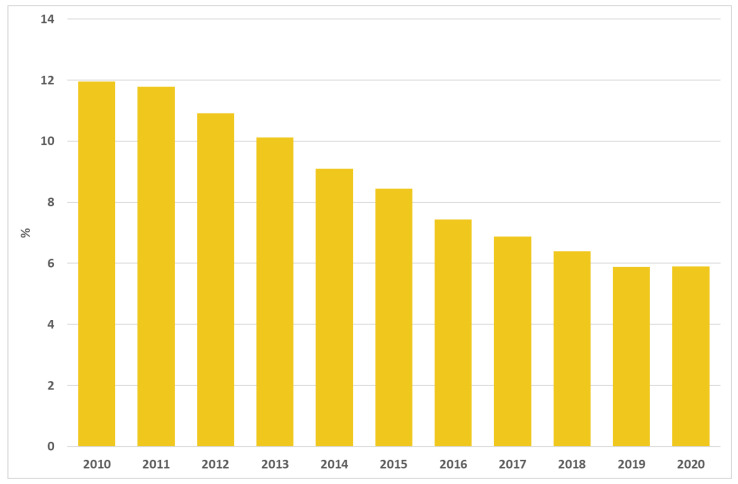
Proportion of reoperation within 90 days of breast-conserving surgery from 2010 to 2020.

A total of 33,135 surgical interventions for breast cancer in 2020 in Italy were analysed; the proportion of reinterventions within 90 days decreased in the last few years, from 12.0% in 2010 to 5.9% in 2020 (Figure 5).

**Figure 6:**
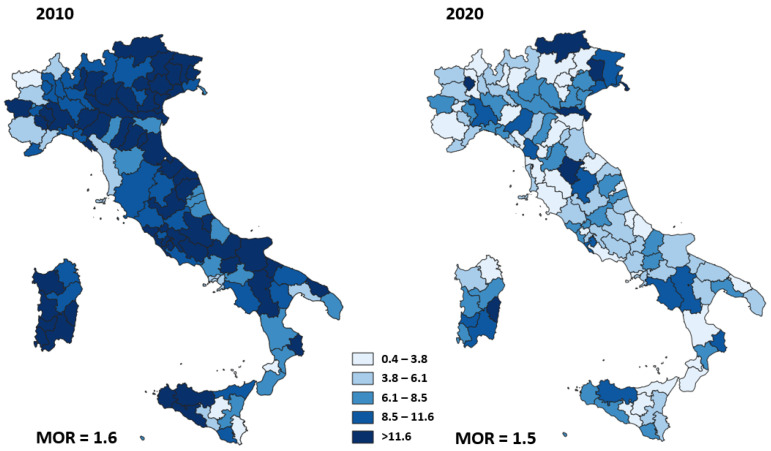
Adjusted proportion of reoperation within 90 days of breast-conserving surgery for Italian area of residence in 2010 and 2020.

Figure 6 shows the adjusted proportion of reintervention within 90 days from breast cancer surgery for each area of residence in 2010 and 2020. The geographic variation of proportion of reinterventions within 90 days ranged from 1.8% to 34.0% (MOR = 1.6) in 2010 and was similar to the geographic variation in 2020, ranging from 0.4% to 18.0% (MOR = 1.5).

**Figure 7:**
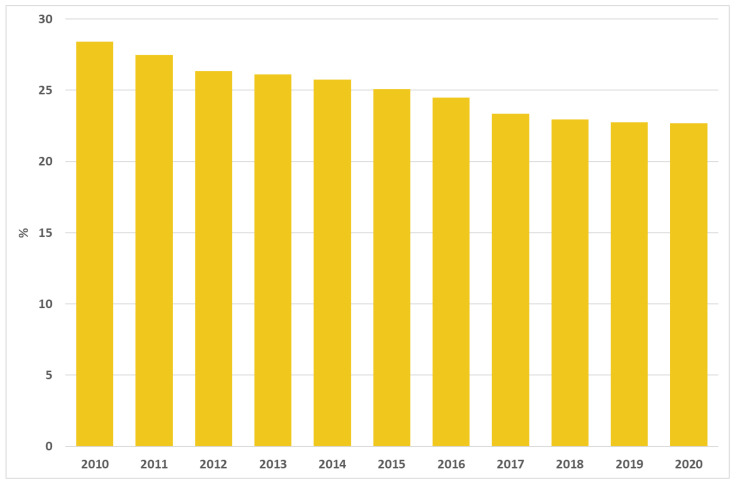
Proportion of primary caesarean deliveries. Italy, 2010–2020.

There were 309,394 deliveries (with no previous C-section) in 2020 and the proportion of primary caesarean deliveries slightly decreased in the last few years, from 28.4% in 2010 to 22.7% in 2020 (Figure 7).

**Figure 8:**
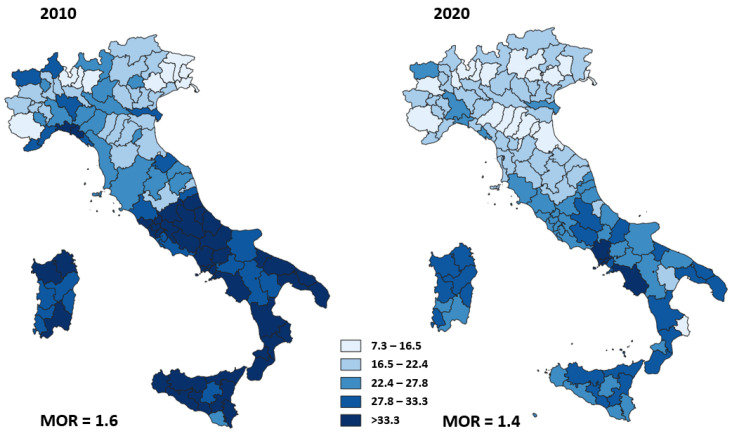
Adjusted proportion of primary caesarean deliveries for Italian area of residence in 2010 and 2020.

Figure 8 shows the adjusted proportion of primary caesarean deliveries for each area of residence in 2010 and 2020. The geographic variation of this proportion ranged from 11.5% to 57.3% (MOR = 1.6) in 2010 and was higher than the geographic variation in 2020, ranging from 7.3% to 40.0% (MOR = 1.4).

## 4. Discussion

The reported results highlight the improvement of some of the most representative performances in the cardiovascular, surgical, orthopaedic and perinatal areas. From 2010 to 2020, we observed a reduction of mortality after AMI and of reoperation within 90 days of breast-conserving surgery, a slight reduction of primary caesarean deliveries and an increased proportion of timely surgery after hip fracture in the elderly. These results suggest the positive impact of comparative evaluation programmes on improving health care quality [4,5,6,7,8]. The availability and public disclosure of P.N.E. results to clinicians, health managers, and policy makers was the basis for the promotion of strategies to improve performances. In fact, public outcome data increase the accountability of providers, stimulate changes at hospital level and promote quality improvement actions in health care organisation [33,34,35].

We observed wide heterogeneity in the proportion of HF surgeries performed within 2 days in 2010. Although this heterogeneity was reduced by 40% in 2020, a high variability of timely surgery still remains. There was a large variability of proportion of primary caesarean deliveries in 2010 that slightly decreased in 2020 while the mortality after AMI and the proportion of reoperation within 90 days of breast-conserving surgery did not show changes in heterogeneity between 2010 and 2020.

Our results have some limitations, especially with regard to the coding accuracy of current health information systems that could have a potential impact on risk adjustment and the reliability of comparative quality ratings [36]. Some studies have reported that changes in data accuracy may partially explain quality improvement [37]. However, there was not a documented different reporting of comorbidities in the years included in our analysis. Moreover, previous Italian studies [38,39] assessing clinical performance in cardiac surgery shows that the use of data from health information systems provided similar ranking as a specialized clinical database. Even though we cannot exclude the possibility of gaming of the data in response to the performance evaluation, previous studies did not demonstrate the evidence of gaming [40].

Furthermore, the large numbers of patients included in our analysis, the accuracy of the selection criteria of the cohorts and outcomes and the validated statistical methodology assure the internal and external validity of the P.N.E. programme. Moreover, the data from hospital information systems can be used to predict outcomes with discrimination comparable with that obtained from clinical databases [41].

Our findings are in line with the results of programs conducted in other countries. The results are similar in terms of general improvement and heterogeneity between structures. Compared to other outcome evaluation programs, the P.N.E. lacks the evaluation and publication of the results at operator level for the outcome and process indicators [3,4,10,11].

## 5. Conclusions

The P.N.E. programme shows a general improvement of outcome indicators in different clinical areas, following the implementation of comparative evaluation at national level. On the other hand, this general improvement of performance is not always associated with a reduction of heterogeneity among areas of residence. Our results underline that P.N.E. can play a critical role in promoting systematic monitoring of quality of health care and stimulating the accountability of health care providers operating within the Italian NHS. Moreover, the P.N.E. could be used as an operative tool for the support of process aimed at reducing heterogeneity of key health performances and improving healthcare equity.

In the next future, the P.N.E. should increasingly become the basis for the development of audit programmes at local level, in order to strengthen interventions for improving health care starting from the critical issues identified. Future studies should be focused on the evaluation of organizational and economic impact of the results of the outcome comparative evaluation programs.

## Figures and Tables

**Table 1:** Milestones of quality improvement in Italy.

Year	Milestone
2010	The first edition of P.N.E. was published.
2012	The Italian Ministry of Health entrusted AGENAS with the national coordination of P.N.E.
2013	The assessment of quality of data recorded in the Health Information System was started in order to evaluate the validity of P.N.E. indicators.
2015	The Italian Ministry of Health defined the quality standards of hospital care according to P.N.E. results *.
2016	The Italian Ministry of Health established improvement programmes for the hospitals with poor performances according to P.N.E. results **.

* Ministerial Decree 2 April 2015, no. 70. ** Ministerial Decree 21 June 2016.

## Data Availability

The data are available from AGENAS with its permission and upon reasonable request, by contacting direzione.ricerca@agenas.it.
